# Bupivacaine Liposomal Injectable Suspension (Nocita^®^) via Transversus Abdominis Plane (TAP) Block for Management of Pain in Dogs with Acute Pancreatitis: A Pilot Study

**DOI:** 10.3390/ani16203272

**Published:** 2026-10-17

**Authors:** Morgaine Angst, Christopher J. Thibault, Sichao Wang, Harry Cridge

**Affiliations:** 1 Department of Small Animal Clinical Sciences, College of Veterinary Medicine, Michigan State University, East Lansing, MI 48824, USA; angstmor@msu.edu (M.A.); thibau11@msu.edu (C.J.T.); 2 Center for Statistical Training and Consulting, College of Veterinary Medicine, Michigan State University, East Lansing, MI 48824, USA; wangsic1@msu.edu

**Keywords:** pancreatitis, analgesia, opioid sparing, local block, regional analgesia

## Abstract

Abdominal pain is one of the most salient features of acute pancreatitis (AP) in dogs. While traditional opioids provide excellent analgesia, they are also associated with significant adverse effects, including ileus. The transversus abdominis plane (TAP) block has been shown to manage pain associated with AP in humans. Ten client-owned dogs with naturally occurring AP were enrolled in a blinded placebo-controlled clinical trial, receiving either a TAP block with bupivacaine liposome injectable suspension (*n* = 5) or traditional opioid analgesia (*n* = 5). Rescue analgesia was administered when indicated based on pain score monitoring every 4 h. Each dog was also administered an imaging device to track gastrointestinal transit time (GITT). The total opioid dose during the 72 h study period was significantly lower in the TAP block group (0.18 ± 0.27 mg/kg) compared to the control (0.99 ± 0.50 mg/kg) group (*p* = 0.01). GITT was prolonged in both the TAP block group (2/3) and the control group (4/5) compared to published references for healthy dogs. Bupivacaine liposomal injectable suspension administered via TAP block may provide sufficient analgesia in dogs with AP while also minimizing their total opioid requirements. Delayed gastric transit was common in both groups, suggesting that factors beyond just opioid administration contribute to gastrointestinal dysmotility in dogs with AP.

## Simple Summary

Acute pancreatitis (AP) is a common disorder in dogs, with abdominal pain being one of the most salient features of this disease. While opioids provide excellent pain control, their adverse effects, including slowing of the digestive system, can impact veterinary patient care. We found that dogs hospitalized with AP that received a local anesthetic treatment with a long-lasting local anesthetic had their pain sufficiently controlled, similarly to dogs receiving traditional opioid pain management. A majority of the dogs in both treatment groups had slow transit times through the stomach, indicating that the slowing of the digestive tract may be due to several contributing factors. Although more research is needed, local anesthesia techniques could help to reduce opioid use in dogs with AP.

## 1. Introduction

Acute pancreatitis (AP) is the most common disorder of the exocrine pancreas in dogs [1]. One of the most salient features of AP is abdominal pain. Other clinical features include inappetence, gastrointestinal upset, and lethargy [2]. Opioids are generally recommended for the analgesic management of dogs with AP [3].

While opioid analgesia is commonly recommended, this approach can be associated with clinically relevant adverse effects, including negative impacts on gastrointestinal transit time (GITT) and the potential for development of ileus. In humans with AP, opioid use has been shown to significantly impact intestinal motility independent of disease severity and other covariates [4]. An additional study found that higher opioid doses during the first 12 h of hospitalization were associated with longer hospital stays and increased cost of care [5].

One potential opioid-sparing technique is the use of local anesthetic drugs and blockade. Local anesthetic agents block pain transmission before the signal reaches the central nervous system, providing profound analgesia, while also providing a lower incidence of dose-related adverse effects [6]. Given this, local and regional anesthetic techniques are growing in popularity and are supported by industry groups such as the 2022 American Animal Hospital Association (AAHA) pain management guidelines [7]. The transversus abdominis plane (TAP) block is a regional analgesic technique that desensitizes the nerves innervating the abdominal muscles, subcutaneous tissue, and parietal peritoneum [8,9]. A canine cadaver study showed that 61–100% of the ventral branches of T9–T13 (which innervate the cranial abdominal wall) were exposed to a dye utilizing an ultrasound-guided TAP block, while largely sparing the caudal abdomen [9]. A prospective interventional study in adult humans with AP found that patients who received a TAP block and opioids to manage pain associated with AP had significantly lower pain scores at 1, 6, 12 and 24 h, lower mean opioid consumption, and lower mean hospital stays compared to those who received opioids only [10]. The TAP block is therefore considered suitable for the management of cranial abdominal pain, and it has been used with success clinically, including one dog with AP and concurrent diabetic ketoacidosis [11]. Many local anesthetic agents can be utilized with the TAP block; however, liposomal bupivacaine (Nocita^®^) has a prolonged duration of action of up to 72 h. This duration of action is similar to the median duration of hospitalization for dogs with AP in large cohort studies [12]. Additionally, liposomal bupivacaine via TAP block has been associated with lower opioid use and improved intestinal function relative to intravenous opioid analgesia in humans undergoing pancreatic transplant [13]. No reported studies to the authors’ knowledge have prospectively evaluated the use of a liposomal bupivacaine TAP block for analgesia in dogs with AP.

The objective of our pilot study was to evaluate the analgesic efficacy of liposomal bupivacaine, administered via ultrasound-guided TAP block in dogs with AP. An additional exploratory aim was to determine the impact of traditional opioid analgesia and the liposomal bupivacaine, administered via TAP block, on intestinal transit time in dogs.

## 2. Materials and Methods

Dogs presenting to a university teaching hospital between August 2024 and March 2026 with suspected AP were eligible for prospective enrollment in the study. The owners of known eligible cases were contacted by study investigators for potential enrollment. The study protocol was approved by the Institutional Animal Care and Use Committee (IACUC) at Michigan State University. Informed consent was obtained from all owners.

The diagnosis of AP was based on integration of clinical signs, pancreatic lipase concentrations, and abdominal ultrasound findings [14]. Dogs were required to have a minimum of two of the following clinical signs: hyporexia, dysrexia, vomiting, lethargy, and/or abdominal dysfunction. In addition, dogs were required to have an abnormal patient-side pancreatic lipase test result (SNAP cPL, IDEXX Laboratories Inc., Westbrook, ME) and a minimum of two of the following ultrasonographic abnormalities: enlarged pancreas, hypoechoic pancreatic parenchyma, and a hyperechoic peri-pancreatic mesentery. Dogs were also required to weigh > 10 kg due to placement of a capsule endoscopy device (ALICAM; Infiniti Medical, Orange City, IA, USA). Once formally enrolled, blood was collected for measurement of canine pancreatic lipase immunoreactivity (PLI) concentration (Spec cPL, Texas A&M Gastrointestinal Laboratory, College Station, TX, USA).

Dogs randomized to the experimental group received a TAP block with bupivacaine liposome injectable suspension (Nocita^®^; Elanco US Inc., Greenfield, IN, USA). A two-site (subcostal and pre-iliac) ultrasound (Sonosite X-Porte; Fujifilm Sonosite Inc., Bothell, WA, USA) guided TAP block was performed on both the left and right hemi-abdominal walls by trained investigators (CJT, MA). A video outlining the technique is provided as Supplementary Material (Video S1). Dogs were sedated with alfaxalone (1–2 mg/kg IV to effect for adequate sedation; Alfaxan multidose; Zoetis Inc., Kalamazoo, MI, USA) and placed in dorsal recumbency. Hair from the abdomen had been previously clipped during the prerequisite abdominal ultrasound, and the abdomen was aseptically prepared. Injectate (5.3 mg/kg liposome-encapsulated injectable suspension, NOCITA, 13.3 mg/mL; expanded to 1 mL/kg with sterile normal saline) was prepared immediately prior to administration. To maintain blinding, a 0.02 mL/kg placebo (saline) was administered IV q4h (equivalent volume to 0.2 mg/kg methadone (10 mg/mL)) for the enrollment period (72 h).

For the subcostal site, a high-frequency (15–6 Hz) linear array transducer (HFL50; Fujifilm Sonosite) was oriented obliquely immediately caudal to the last rib and placed as abaxial as possible from midline while visualizing the abdominal wall landmarks. At this site, the three muscle layers of the abdominal wall (from superficial to deep), external abdominal oblique, internal abdominal oblique and transversus abdominis muscles, were visualized. The target area for local anesthetic infiltration was defined as immediately deep to the fascia located on the surface of the transversus abdominis plane [15]. A 21 g × 80 to 100 mm insulated needle (EchoStim; Havel’s LLC, Cincinnati, OH, USA) was introduced in plane with the puncture site approximately at the costochondral junction. Needle length was selected based on the enrolled dogs’ size. Correct placement of the needle tip was confirmed by visualizing the hydrodissection of the correct plane with a small test dose of injectate (0.5–1 mL) before injecting 0.25 mL/kg of the total dose. This process was repeated for the pre-iliac site, with the transducer oriented in the craniocaudal direction just cranial to the iliac crest (puncture site). These processes were then repeated on the contralateral side of the abdomen for a total volume of 1 mL/kg instilled. All dogs in this group recovered uneventfully from sedation.

Dogs randomized into the control group received methadone (0.2 mg/kg, methadone hydrochloride injection, USP; Mylan Institutional LLC, Morgantown WV) IV q4h for the 72 h enrollment period and received no TAP block.

Both groups were evaluated for pain using the short-form Glasgow Composite Pain Scale (sf-GCPS) [16] q4h on the opposite schedule as the methadone or saline boluses. A video outlining how to perform the sf-GCPS is provided as Supplementary Material (Video S2). If any dogs were found to have a pain score from the sf-GCPS of 6 or higher, a 0.1–0.2 mg/kg methadone IV bolus was administered as rescue analgesia. Pain scoring was performed by licensed veterinary technicians working in the emergency and critical care department of the university teaching hospital after training on the sf-GCPS. Veterinary technicians were blinded to the dogs’ treatment group, and both the methadone and saline administered q4h were drawn up by one of the investigators (MA), kept in a lock box assigned to the enrolled dog, and labeled “Blinded Analgesic Treatment”. The blinded analgesic treatment was administered to the dogs for 72 h while in hospital, or until discharge from the hospital, or removal from the study protocol based on the judgement of the attending veterinarian. Alongside analgesia, enrolled dogs received a standardized treatment protocol for AP (Table 1). All other treatments deemed medically necessary by the attending veterinarian were permitted, and other treatments were recorded.

Ambulatory light-based imaging devices (ALICAM; Infiniti Medical, Orange City, IA, USA) were administered to each enrolled dog per os to image and track transit times between different sections of the gastrointestinal tract. Bravo pH motility capsules were unavailable at the time of the study. Following collection, imaging devices were shipped for analysis, and detailed reports were returned.

## 3. Statistical Methods

Baseline characteristics of the TAP block and control (traditional opioid analgesia) groups were compared using Pearson’s Chi-squared tests and Wilcoxon matched pairs tests as appropriate. Descriptive analyses were also performed. Significance for all statistical comparisons was set at *p* < 0.05.

## 4. Results

### 4.1. Animals

Ten dogs were prospectively enrolled in the study. Five dogs were randomized to the TAP block group and five to the control (traditional opioid analgesia group). The demographics of the enrolled dogs, including age, weight, sex, and PLI, were not significantly different between groups (Table 2).

### 4.2. Analgesia

All five dogs in the TAP group received the local block without complications. Two of the five dogs in the TAP group required at least one rescue analgesic (methadone) dose during the 72 h of the study. The rescue doses were required at 34, 39, 47, 59, and 62 h after TAP block in one dog, and at 1, 16, and 20 h after TAP block in the second dog.

Two of five dogs in the control (traditional opioid analgesia) group required at least one rescue methadone analgesic dose. The total methadone dose during the study was significantly lower in the TAP block group (0.18 ± 0.27 mg/kg) compared to the control (traditional opioid analgesia) (0.99 ± 0.50 mg/kg) group (*p* = 0.01).

### 4.3. Gastrointestinal Motility Assessment

Of the 10 ALICAMs administered, eight were recovered following fecal passage and were submitted for analysis. Two cameras were passed at home after discharge but were not recovered (lost).

Of the five dogs in the TAP group, two ALICAMs were not recovered. Both cameras were noted radiographically within the gastrointestinal tract during hospitalization but were not witnessed by the owner to have been passed following discharge. Both dogs had clear abdominal radiographs at a recheck examination confirming successful passage of the device. As the devices were not collected, GITT was not available for these dogs. Of the three dogs with data available, all had normal (rapid) esophageal transit. Two dogs had delayed gastric motility, and capsules were noted to remain within the stomach for the duration of capsule recording (48–59 h). One dog was noted to have normal gastric and small intestinal transit. Two of the three dogs were noted to have gastric or duodenal ulceration, one of which was clinically evident and was managed with a proton pump inhibitor.

Of the five dogs in the control (traditional opioid analgesia) group, all devices were recovered. All five dogs had normal (rapid) esophageal transit. Four of five dogs had delayed gastric emptying, and capsules were noted to remain within the stomach for the duration of capsule recording (69–220 h). One dog was noted to have accelerated gastric and small intestinal transit. No dogs in this group received a prokinetic. All five dogs were noted to have gastric ulcerations or erosions, none of which was clinically evident.

### 4.4. Additional Treatment Notes

Two of five dogs in the TAP block group received gabapentin following the 72 h study period (12.9–17.4 mg/kg PO q8h. Three of five dogs in the control group received gabapentin following the 72 h study period (8.4–14.4 mg/kg PO q8h).

Ondansetron (0.2–0.5 mg/kg IV q8h) was required in all 10 dogs to assist in management of vomiting and/or nausea. A proton pump inhibitor (esomeprazole 1 mg/kg IV q12h) was prescribed to one dog in the study (in the TAP block group) due to clinically evident gastrointestinal bleeding, noted at study enrollment.

Two of five dogs in the TAP block group ate without the need for nasogastric tube feeding, compared to no dogs in the control (traditional opioid analgesia) group.

Three dogs (two control, one experimental) received fuzapladib sodium (Panoquell-CA1) prior to their presentation and subsequent hospitalization, and one dog (control group) received the drug in hospital after the study period.

## 5. Discussion

In this pilot study, we were able to document successful performance of an ultrasound-guided TAP block in five dogs with AP. The TAP blocks were performed without complication by a trained individual.

Bupivacaine liposome injectable suspension currently holds FDA approval for post-operative analgesia in dogs undergoing cranial cruciate ligament surgery and in cats following onychectomy [6]. The prolonged release technology is due to multivesicular liposomes encapsulating aqueous bupivacaine. The vesicles gradually release bupivacaine over 96 h as lipid bilayers break down. As bupivacaine is released from individual liposomes, it is distributed locally to surrounding tissues and eventually into systemic circulation [18]. The analgesic properties of bupivacaine liposome injectable suspension have been investigated in other applications, including abdominal surgeries. One study comparing the need for rescue analgesia in dogs undergoing ovariohysterectomy found that dogs that received bupivacaine liposome injectable suspension were less likely to require rescue analgesia and had lower pain scores compared to those receiving a bupivacaine splash block [19]. Another study concluded that bupivacaine liposome injectable suspension had comparable pain control to opioids alone in dogs undergoing abdominal surgeries [20].

In this study, the TAP block was performed using the subcostal and pre-iliac sites to ensure adequate cranial, caudal and ventral spread of the bupivacaine liposome injectable suspension to produce sensory blockade to the spinal nerves innervating the abdominal wall. The TAP block has long been used for somatic pain as it provides sensory blockade to the abdominal wall and associated tissues. However, some studies have suggested the technique’s utility in providing analgesia for visceral pain in humans and dogs [10,11]. While the mechanism for providing visceral analgesia is still unclear, it has been postulated that this is an indirect effect through the blockade of overlapping thoracolumbar afferent pathways and modulation of central sensitization [10]. Significant overlap of somatic and visceral afferents in the dorsal horn of mammalian species has been demonstrated, suggesting the distinction between somatic and visceral pain may not be as clear as previously thought [21,22]. Alternatively, a second proposed mechanism suggests that the analgesic effects of the TAP block may partially depend on the rise in serum concentration of the local anesthetic [23]. While this study adds to the data supporting the utility of the TAP block for control of visceral pain in inflammatory conditions, further investigation into this mechanism is needed.

We were also able to demonstrate that the bupivacaine liposomal injectable suspension TAP block provided sufficient analgesia in three of five dogs, as evidenced by the lack of requirement for rescue analgesia based on serial monitoring of the sf-GCPS. Two dogs in the TAP group and two dogs in the control (traditional opioid analgesia) group required rescue analgesia, emphasizing the need for careful monitoring of pain scores in affected dogs and responding accordingly with additional analgesia. The time to rescue analgesia varied in the two dogs receiving the TAP block, with one dog requiring rescue analgesia as early as 1 h after intervention. This could represent either treatment failure or a delayed onset of action. This dog required no additional rescue analgesia until 15 h later, suggesting the potential for delayed analgesic efficacy. The second dog did not require rescue analgesia until 34 h after TAP block but then required rescue analgesia fairly regularly, suggesting a ‘wear-off’ effect.

The differences in the time to rescue analgesia between these dogs exemplify the sparse information currently available on the onset and duration of action of bupivacaine liposome injectable suspension and the TAP locoregional technique. The onset of action is 2–5 min in humans but has not been reported in animals. While this drug is labeled to provide analgesia for up to 72 h in dogs undergoing cranial cruciate ligament surgery, it is important to note that some dogs in that study were painful before 72 h had elapsed [24]. Information on the duration of the TAP block is scarce [15]. Generally, the duration of sensory blockade is thought to be dependent on the local anesthetic instilled into the fascial plane; however, more clinical studies are needed. One study showed that the use of a TAP block resulted in lower pain scores and a lower need for rescue analgesia in dogs undergoing ovariohysterectomies compared to a placebo; the addition of bupivacaine liposome injectable suspension did not provide any additional benefit compared to a TAP with bupivacaine and dexmedetomidine [15].

The dogs in the TAP block group also received a significantly lower total dose of methadone compared with the control group. Although this may be anticipated given the study design, it provides additional support for the analgesic efficacy of the bupivacaine liposomal injectable suspension TAP block. Specifically, inadequate analgesia would have necessitated additional methadone administration (rescue dosing), resulting in more similar opioid requirements between the two groups.

An exploratory aim was to evaluate the impact of both traditional opioid analgesia and the liposomal bupivacaine TAP block on intestinal transit time in dogs. Opioids are considered the mainstay of analgesia in pancreatitis, but they offer significant limitations, most notably the potential for ileus [3]. While the potential for ileus is well recognized, there is no prospective data on the impact of analgesic choice on gastrointestinal motility in dogs with AP. Given that opioid use has been shown to significantly impact intestinal motility independent of AP severity and other covariates and that higher opioid use has been associated with prolonged hospitalization in humans, we hypothesized that use of non-opioid analgesia, such as the liposomal bupivacaine TAP block, would result in faster gastrointestinal motility [4,5].

Several methods have been described to study gastrointestinal motility in dogs. Radioscintigraphy is considered the gold standard method to quantify gastrointestinal transit time, but it is rarely utilized due to the need for specialized equipment and radiation licensing [25]. In the absence of radioscintigraphy, wireless capsule monitoring techniques are considered a suitable alternative. A continuous pH monitoring system (Bravo pH monitoring system; Medtronic), which determines location within the gastrointestinal tract based on pH, was initially considered for the study, but these devices were unavailable at the time of study enrollment [26,27]. Video capsule endoscopy (VCE) (Alicam, Infiniti Medical, Redwood City, CA, USA) was used in place of the pH capsule in the present study. The Alicam device is a camera device propelled through the gastrointestinal tract via peristalsis. As the device passes through the gastrointestinal tract, it obtains images, which can then be used to determine the capsule location and measure time between images based on the internal clock [28]. Gastrointestinal segment transit time can be compared to published parameters for healthy dogs to determine if motility is accelerated, normal, or prolonged. The median gastric transit time for fasted healthy dogs is 86 min (range: 13–218 min), while the median small intestinal transit time is 124 min (range 34–168 min).

In our study, gastric transit time was prolonged in six of eight dogs with available data, including dogs in the traditional opioid analgesia (69–220 h, *n* = 4/5) and TAP block (48–59 h, *n* = 2/3) groups. Due to the low statistical power from the small sample size, the loss of two capsules following discharge, and the non-standardized duration of recording, statistical comparison between groups was not performed, and GITT findings are presented descriptively. Even so, the magnitude of delay relative to healthy fasted dogs (median 86 min) is clinically relevant. While many clinicians attribute delayed gastrointestinal motility to opioid administration, prolonged gastric transit occurred in both groups, including dogs receiving an opioid-sparing technique. This suggests that gastrointestinal dysmotility in dogs with AP is multifactorial. We propose that the inflammatory process of AP itself, including local peritonitis adjacent to the stomach and duodenum, may be a primary contributor, with additional effects from enteral nutrition, hospitalization, electrolyte abnormalities, opioid administration, gastrointestinal ulceration and individual animal factors. The present data cannot determine the relative contribution of each factor. A larger study with standardized recording duration and in-hospital capsule retention is needed to test these hypotheses and evaluate interactions between opioid dosing and gastrointestinal motility in dogs with AP.

While not a specific objective of our study, it is notable that several dogs in our study had clinically silent gastrointestinal ulcerations noted on VCE. Until recently, acid-suppressant medications were often utilized in dogs with pancreatitis; however, the 2018 ACVIM consensus statement on the rational use of gastroprotectants in dogs and cats highlighted the lack of evidence to support their use and prompted a shift in this practice [29]. Given the unanticipated high frequency of visible mucosal ulcerations on VCE, additional studies are needed to determine the frequency and clinical significance of these abnormalities. Such studies would help to inform rational prescribing practices in dogs with AP.

Our pilot study had several limitations, including a small sample size, which limited statistical power. Two video capsules were passed at home following discharge and were not recovered, and the duration of capsule recording was not standardized, further limiting the GITT data. Future studies should retain dogs in hospital, or arrange supervised collection, until capsule passage is confirmed. Although analgesic efficacy was based on validated scoring systems, these measures remain somewhat subjective and may be influenced by factors such as stress and other aspects of hospitalization. Additionally, for ethical reasons, dogs received a variety of supportive therapies alongside their analgesic treatment, limiting our ability to isolate the effects of the analgesic intervention on study results. These limitations should be considered when interpreting the findings of this pilot study.

## Figures and Tables

**Table 1:** Treatment Protocols.

	TAP Block Group	Control Group
Standard Analgesia	TAP block.	Methadone: 0.2 mg/kg q4h.
Rescue Analgesia	Methadone: 0.1 mg/kg up to every 4 h (if CMPS-sf ≥ 6/24).	
Fluid Therapy	2× BSAVA (40–60 mL/kg/day) [17] maintenance fluid rate of LRS. Adjusted to maintain euhydrated status.	
Standard Anti-emetic	Maropitant Citrate: 1 mg/kg IV q24h.	
Rescue Anti-emetic	Ondansetron: 0.5 mg/kg IV (slow) q8h.	
Enteral nutrition	Eating or (if ≥3 days of inappetence despite supportive care) nasogastric (NG) tube * feeding: Royal Canin GI Low Fat Liquid Diet (Day 1: 1/4th RER, day 2: 1/3rd RER, day 3: 1/2 RER, if tolerated).	
± Ancillary therapies (covered by client)	Electrolyte supplementation: KCl, KPhos as needed. Gastric acid suppression: pantoprazole 1 mg/kg IV q12h only if there is clinical evidence of gastrointestinal ulceration (hematemesis or melena) or esophagitis. Fresh frozen plasma transfusion: only if documented clinically significant coagulopathy. Antibiotics: only if pancreatic abscessation, concern for bacterial translocation, or if a secondary complication (e.g., aspiration pneumonitis) warrants antimicrobial therapy. Antibiotic selection at discretion of attending clinician. Glucocorticoids: prednisolone 0.5–1.0 mg/kg/d only if dog fails to respond to aggressive supportive care of 48 h duration. Fuzapladib sodium: permitted at clinician discretion.	

* Alfaxalone 2.5 mg/kg IM will be utilized for NG tube placement and/or the TAP block.

**Table 2:** Demographics of Enrolled Dogs.

	Control *N* = 5	Experimental *N* = 5	Test Statistic
Age (years)	9.0 ± 1.6	8.6 ± 4.0	*p* = 0.85
Weight (kg)	27.8 ± 11.6	20.8 ± 10.5	*p* = 0.38
Breed	Mixed breed (3) Australian shepherd (1) Doberman pinscher (1)	Pug (2) Golden retriever (1) Mixed breed (1) Springer spaniel (1)	N/A
PLI (µg/L)	1408 ± 825	950 ± 806	*p* = 0.37
Sex	Male castrated (3) Female spayed (2)	Male castrated (4) Female spayed (1)	*p* = 0.49

Age, weight, and PLI concentration are reported as mean ± 1 standard deviation from the mean.

## Data Availability

As the study involved client-owned animals, the underlying clinical data is subject to privacy and confidentiality restrictions. Further data may be available from the corresponding author upon reasonable request, subject to appropriate permissions and privacy considerations.

## Supplementary Materials

The supporting information: Video S1: Performing an ultrasound-guided Transversus Abdominis Plane (TAP) block on an anesthetized canine patient. Link: https://zenodo.org/records/22261545?token=eyJhbGciOiJIUzUxMiJ9.eyJpZCI6ImJmYWJkNDRkLWUwNmYtNDRmZC1hODRjLWRhZjRjNzVjODY4MyIsImRhdGEiOnt9LCJyYW5kb20iOiIyODg2YmExMzA0MjU5NWMwYTE0YTgwMDA3OTk5MWE2ZiJ9.RRbojAejZbkPzL1_0ZOrBn0iU33k9b6bqTrdqEOYWLlIkiqZoCSEFksLMSowHEiCoY84rU6prKnbZ4KHVJaAvw (accessed on 10 May 2026); Video S2: Use of the short form Glasgow Modified Pain Scale (sf-GMPS) to assess acute pain in a dog. Link: https://zenodo.org/records/22260642?token=eyJhbGciOiJIUzUxMiJ9.eyJpZCI6ImI2NDE2NzdmLWNmZTQtNGQxMi1hOWU5LTI1MjkyOTRiNzkyMCIsImRhdGEiOnt9LCJyYW5kb20iOiI3MTlhZWFjNjI4ODUyZWRmYTE4ZDA4MTVkMWI2MmQ0MiJ9.BBHdcqVGy8jyu_be4oGVf7-7L7ntSacIzhqB0j-yER5jVZkkQOtvD0u16pXDkBbjeECiqYTxYqkmNI3WLENiKQ (accessed on 10 May 2026).

## Abbreviations

The following abbreviations are used in this manuscript:

AP	Acute pancreatitis
IACUC	Institutional Animal Care and Use Committee
NG	Nasogastric
NSAID	Non-steroidal anti-inflammatory drug
PLI	Pancreatic lipase immunoreactivity
RER	Resting energy requirement
Sf-GCPS	Short form Glasgow Composite Pain Score
Spec cPL	Canine pancreatic lipase immunoreactivity concentration
TAP	Transversus abdominis plane
VCE	Video capsule endoscopy
